# Parental Ease in Asking Others Not to Smoke and Respiratory Symptoms and Illness among Children

**DOI:** 10.3390/ijerph110201747

**Published:** 2014-02-05

**Authors:** John Spangler, Zsuzsanna Csákányi, Todd Rogers, Gábor Katona

**Affiliations:** 1 Department of Family and Community Medicine, Wake Forest School of Medicine, Wake Forest University, Winston-Salem, NC 27157, USA; E-Mail: katonagbor@gmail.com; 2 Department of Otorhinolaryngology, Heim Pal Children’s Hospital, ENT Department Üllói út 86. H-1089 Budapest, Hungary; E-Mail: csakanyi.zsuzsanna@gmail.com; 3 RTI International, 351 California Street, Suite 500, San Francisco, CA 94104, USA; E-Mail: trogers@rti.org

**Keywords:** tobacco smoke, respiratory illness, children, Hungary

## Abstract

*Objective*: Childhood exposure to secondhand tobacco smoke (SHS) increases a child’s burden of respiratory conditions, but parental smoking bans may reduce such morbidity. This study evaluated household smoking bans and their relationship to respiratory illness in an outpatient otolaryngology clinic. *Methods*: The study was performed at the Heim Pal National Children’s Hospital, Ear, Nose and Throat (ENT) Department (Budapest, Hungary) from July to November, 2010. A consecutive series of children’s caregivers were approached to participate in a survey measuring household smoking bans, upper and lower respiratory tract symptoms and illnesses, and socioeconomic factors. Bivariate and multivariate logistic regression analyses were performed. *Results*: Of the 215 caregivers recruited for the study, 208 agreed to participate (response rate of 96.7%). More than half of the children were male (54%), and 39% lived in a household with at least one member who smoked. Smoking was frequently banned inside the car (91.3%) and home (85.1%). Respondents felt it easiest to ask friends (97.1%) and family members not living in the household (98.1%) to refrain from smoking inside the home. Respondents also found it easier to ask a stranger (81.7%) or a family member (61.1%) not to smoke around the child. Logistic regression showed that respondents for children with a history of pneumonia found it less difficult to ask visitors in the home not to smoke compared to children without pneumonia (OR = 0.23, 95% CI = 0.06–0.98). Conversely, respondents for children who had had adenoidectomy found it over three times more difficult to ask strangers not to smoke near the child compared to those of children without adenoidectomy (OR = 3.20, 95% CI = 1.43–6.38). *Conclusions*: In a population of children visiting an outpatient ENT clinic in Budapest, Hungary, we found a high degree of exposure to SHS. The ease with which caregivers felt towards asking others not to smoke predicted specific respiratory conditions. Since the ENT clinic offers a wonderful opportunity for clinicians to counsel parents on tobacco cessation, increased tobacco education of these providers is needed.

## 1. Introduction

Exposure to second hand tobacco smoke (SHS) is hazardous and associated with striking morbidity and mortality from cancer, heart disease, adverse infant and perinatal outcomes, and respiratory illnesses [1]. The dangers of SHS are particularly serious for children, increasing their vulnerability to sudden infant death syndrome, otolaryngologic conditions such as otitis media, and lower respiratory diseases, and other illnesses [1,2]. A number of factors make children particularly defenseless against SHS exposure [1,2]. For example, children’s ventilation rates are higher than those of adults. Because of their smaller size, they are therefore exposed to proportionally more toxins. Furthermore, unlike adults, children cannot choose to live with non-smokers. They cannot enforce smoke-free bans in private spaces such as the home or the car, nor can they readily leave areas where smokers are currently smoking [2]. Thus, over the past decade, measured cotinine (a nicotine metabolite) levels in children have not declined as rapidly as cotinine levels in adults [1]. While voluntary community interventions have been shown to reduce children’s exposure to SHS in cars and at home [3,4], policy makers [5] and landlords [6] are frequently reluctant to institute outright bans in these microenvironments. Physicians can play a significant role in encouraging protection of children from SHS exposure [7,8,9,10]. Indeed, among pediatric otolaryngologists, the opportunity exists to intervene with smoking parents to reduce children’s contact with SHS [10]. Furthermore, compared to other European countries, children in Hungary have relatively high rates of SHS exposure (40%–50%) [11]. In order to evaluate parental policies of exposure of children to SHS, we carried out this study in a pediatric otolaryngology clinic. We specifically sought to determine not only what policies parents put in place, but also the ease or difficulty of parents’ asking others not to smoke and respiratory illness.

## 2. Methods

The study was performed at the Heim Pal National Children’s Hospital, Ear, Nose and Throat (ENT) Department (Budapest, Hungary) from July to November, 2010. This clinic functions as both a referral otolaryngology clinic for primary care physicians, as well as a clinic to which patients can self-refer. 

A questionnaire was constructed for the parents or caregivers of all children between 6 months and 18 years old presenting for their first visit to the clinic. The questionnaire ascertained demographic information, as well as reported upper and lower respiratory symptoms (e.g., rhinorrhea, nasal congestion, daytime cough, *etc.*), diseases (e.g., middle ear infection, bronchitis, pneumonia, eczema or hay fever), and surgery (e.g., adenoidectomy). Standardized assessment of SHS exposure was also elicited [1]. Parents or caregivers were asked about SHS exposure policies at home, in the car, and near the child. 

During two of the investigators’ (Zsuzsanna Csákányi, Gábor Katona) clinic, a convenience sample of 215 consecutive patients were approached; seven parents or caregivers declined to participate, yielding 208 participants for a response rate of 96.7%. Bivariate and multivariate logistic regression analyses were performed using SPSS version 19 (IBM, Armonk, NY, USA). In logistic regression, the dependent variables were the disease conditions, while the independent predictor variables included parental ease/difficulty in asking others not to smoke. All models also controlled for patient age, gender, size of home, maternal education and parental employment. Significance was set at *p* < 0.05. The institutional review board of Heim Pal approved the protocol.

## 3. Results

The average age of the sample was approximately 72 months. About 54% of the children were male, and 39% lived in a household with at least one member who smoked (Table 1). The most common ENT symptoms were rhinorrhea, nasal congestion and blowing of the nose. Smoking was more frequently banned inside the car (91.3%) and home (85.1%), but slightly less frequently banned around the child while not at home (81.7%).

**Table 1:** Characteristics of the sample with frequency of rhinitis and sinusitis symptoms and parental policies regarding children’s exposure to second hand smoke.

Characteristic	N (%)
Male	113 (54.3)
Households with smokers	82 (38.9)
***Nasal congestion***	
None	27 (13.0)
Mild	63 (30.3)
Moderate	98 (47.1)
Severe	20 (9.6)
***Blowing nose***	
None	19 (9.1)
Mild	34 (16.3)
Moderate	126 (60.6)
Severe	29 (13.9)
***Rhinorrhea***	
None	22 (10.6)
Mild	48 (23.1)
Moderate	125 (60.1)
Severe	13 (6.3)
***Thick nasal discharge***	
None	85 (40.9)
Mild	63 (30.3)
Moderate	51 (24.5)
Severe	9 (4.3)
***Daytime cough***	
None	28 (13.5)
Mild	72 (34.6)
Moderate	81 (38.9)
Severe	27 (13.0)
***Problems sleeping due to symptoms***	
None	84 (40.4)
Mild	63 (30.3)
Moderate	50 (24.0)
Severe	11 (5.3)
***Ear stuffiness***	
None	91 (43.8)
Mild	64 (30.8)
Moderate	38 (18.3)
Severe	15 (7.2)
Middle ear infections	108 (51.9)
Bronchitis	61 (29.3)
Pneumonia	29 (13.9)
Adenoidectomy	49 (23.6)
Punction	7 (3.4)
Asthma	20 (9.6)
FESS	1 (0.5)
Eczema	25 (12.0)
Hayfever	15 (7.2)
Nasal Steroids	36 (17.8)
Antihistamines	41 (19.7)
Antibiotics	141 (67.8)
Daycare	147 (70.7)
Multiunit Housing	46 (22.1)
***Smoking inside home***	
Not allowed	177 (85.1)
Allowed in some places	20 (9.6)
Allowed anywhere	11 (5.3)
***Smoking in car***	
Not allowed	190 (91.3)
Sometimes allowed	12 (5.8)
Always allowed	6 (2.9)
***Smoking around child***	
Not allowed	170 (81.7)
Sometimes allowed	36 (17.3)
Always allowed	2 (1.0)
***Smoke exposure of child at home***	
None	172 (82.7)
A little	27 (13.0)
Moderate	5 (2.4)
Strong	4 (1.9)
Age in months (+SD)	71.6 (41.0)

In general, respondents reported that it was “very easy” or “easy” to ask visitors, friends and family members not to smoke inside their homes (Table 2). Specifically, it was easiest for respondents to ask friends (97.1%) and family members not living in the household (98.1%) to refrain from smoking inside. Regarding requests of others nearby, respondents found it was “very easy” or “easy” or to ask a stranger (81.7%) or a family member (61.1%) not to smoke around the child.

**Table 2:** Difficulty asking people not to smoke inside home or nearby.

**Difficulty asking people not to smoke inside home **	**N (%)**
**Person asked not to smoke**	
*Visitor*	
Very easy/easy	194 (93.3)
Difficult/very difficult	614 (6.7)
*Friend*	
Very easy/easy	202 (97.1)
Difficult/very difficult	6 (2.9)
*Family member not living in household*	
Very easy/easy	204 (98.1)
Difficult/very difficult	4 (1.9)
*Family member living in household*	
Very easy/easy	196 (94.2)
Difficult/very difficult	12 (5.8)
**Difficulty asking people not to smoke near you**	**N (%)**
**Person asked not to smoke near you**	
*Stranger*	
Very easy/easy	170 (81.7)
Difficult/very difficult	38 (18.3)
**Someone you know (not friend or family)**	**N (%)**
Very easy/easy	77 (37.0)
Difficult/very difficult	131 (63.0)
*Friend*	
Very easy/easy	93 (44.7)
Difficult/very difficult	115 (55.3)
*Family member*	
Very easy/easy	127 (61.1)
Difficult/very difficult	81 (38.9)

Multivariate linear regression revealed that the difficulty which respondents felt asking others not to smoke positively correlated with the presence of ENT symptoms (data not shown). Nasal congestion and problems sleeping from ENT symptoms correlated with difficulty asking acquaintances not to smoke (*p* = 0.015 and *p* = 0.008, respectively). Nasal congestion and rhinorrhea correlated with difficulty asking strangers not to smoke (*p* = 0.018 and *p* = 0.032, respectively). Further, the total number of reported ENT symptoms correlated with difficulty asking household members not to smoke (*p* = 0.015).

Predictors of medical conditions are listed in Table 3. Males were more likely to have middle ear infections (Odds ratio (OR) = 1.82; 95% confidence interval (CI) = 1.05–3.17). Children with bronchitis were more likely to be younger (OR = 0.98; 95% CI = 0.98–0.99). Respondents for children with pneumonia found it less difficult to ask visitors in the home not to smoke compared to children without pneumonia (OR = 0.23, 95% CI = 0.06–0.98). Conversely, respondents for children who had had adenoidectomy found it over three times more difficult to ask strangers not to smoke near the child compared to those of children without adenoidectomy (OR = 3.20, 95% CI = 1.43–6.38). Eczema and hay fever correlated with father’s part time employment (compared to employment).

**Table 3:** Logistic regression predictors of medical conditions among children *.

Condition	Predictor	Odds Ratio	95% CI	*p*-value
Middle ear infections	Male sex	1.82	1.05–3.17	0.033
Bronchitis	Age in months	0.98	0.98–0.99	0.002
Pneumonia	Difficult ** to ask visitors not to smoke in home	0.23	0.06–0.98	0.047
Adenoidectomy	Difficult ** to ask strangers to not smoke nearby	3.20	1.43–6.38	0.004
Eczema	Father part time employed (compared to unemployed)	2.89	1.12–7.41	0.028
Hayfever	Age in months	1.01	1.001–1.02	0.031
Parental Employment	Father part time employed (compared to unemployed)	3.35	1.07–10.5	0.038

Notes: * Models were run separately for each condition and controlled for patient age, gender, size of home, maternal education and parental employment. ** “Difficult” combines difficult/very difficult responses.

## 4. Discussion

In an outpatient pediatric otolaryngology clinic, we have found that parents frequently have policies banning smoking inside the home (85%), car (91%) and nearby the child (82%). The corollary to this finding is that up to 28% of parents allow their children to be exposed to SHS. To our knowledge, this is the first pediatric ENT study to ask specifically about parental SHS policies in children’s microenvironments. 

Parents reported that it is easier to ask people not to smoke inside their home compared to asking people not to smoke nearby their children. Generally, individual ENT symptoms as well as the total number of ENT symptoms in children correlated with increased parental difficulty asking household members, acquaintances and strangers not to smoke (data not shown). Furthermore, a history of pneumonia was 77% less likely if parents found it easier to ban smoking inside the home; and adenoidectomy was more over three times more likely if parents found it difficult to ask strangers not to smoke near their children. Ease or difficulty of asking others not to smoke around the child is likely an indirect measure of SHS exposure. Nonetheless, a priori, in an ENT clinic setting these findings would not necessarily be predicted without empirical testing. Moreover, we feel that parental ease/difficulty in asking others not to smoke measures more than simply SHS exposure; for example it might indicate a parent’s concern for his/her child to be uniquely susceptible to the effects of SHS. Still, our SHS findings are important information to share with parents, and to our knowledge have not been previously demonstrated in a pediatric ENT clinic setting. Parental knowledge of harm can increase motivation for them to quit smoking. [1] The association of males with middle ear disease has been previously reported, and is not unexpected. [1] 

The association of eczema and hay fever to fathers’ part time employment status at first seems perplexing, but we have previously shown in this clinic population that fathers’ part time employment status is related to father’s smoking [11]. This might help to explain why these atopic diseases are associated with father’s employment, which has been shown in some, but not all, studies [1]. An additional potential explanation is that part time employment may expose fathers to unique allergens that unemployed and full-time employed fathers are not exposed to. In that scenario, children might be exposed to these allergens from contact with their father’s hair, skin or clothing.

These findings have a number of implications. First, it is important for pediatric otolaryngologists to inquire about children’s exposure to SHS, and to counsel parents to protect them. This is strongly in the interest not only of children’s respiratory health, but their overall health as well. Such counseling, however, will likely require increased training of otolaryngologists during residency in tobacco intervention [10]. Of course, the need for increased medical education in tobacco cessation is not limited to otolaryngologists. In fact, all physicians and medical students need such training [12,13]. Tobacco intervention education is most effective if carried out in multiple settings and if multiple interactive educational methods are used—not simply didactic lectures [12]. Furthermore, these findings should be shared with parents to point out to them the potential harm SHS can cause [1]. In addition, such findings can support local, regional and national efforts to enact policy regarding SHS. Such interventions in these locations have shown significant reductions in the public’s exposure to SHS [1].

This study is subject to a number of limitations. First, symptoms and diagnoses of upper and lower respiratory tracts conditions were self-reported by care givers. While this can introduce error in measurement, the questionnaire used in this study gave examples of these conditions and symptoms to the respondents when asking the questions. Moreover, for many childhood conditions such as otitis media, parental report is acceptably accurate, [13,14] and, in any case, misclassification biases towards then null [13,14]. Generalizability of these data might also be a concern because participants were non-randomly sampled from one pediatric otolaryngology clinic in the Budapest. However, the fact that these findings on children’s SHS exposure reflect other national studies in Hungary diminishes this limitation to some degree [15]. Finally, our study did not use validated questionnaires, although we modeled our questions based on the Surgeon General’s report on SHS [1].

## 5. Conclusions

In a population of children visiting an outpatient ENT clinic in Budapest, Hungary, we found that parental ease in asking others not to smoke around their children is directly correlated with the health of these children. Importantly, children of parents who feel it is easier to ask for no smoking at home and in the car; and those whose parents find it easier to ask others not to smoke near their children, have lower rates of upper and lower respiratory illness. Since the ENT clinic offers a wonderful opportunity for clinicians to counsel parents on tobacco cessation, increased education of these providers in tobacco intervention is needed. Moreover, legislative and voluntary efforts to reduce childhood SHS exposure in the microenvironment have shown to be effective. A comprehensive individual and population level approach to reducing children’s exposure to SHS is most likely to succeed. This should include training medical students and residents [11] and ENT surgeons [10] in how to help parents to stop smoking. 
